# Resveratrol Ameliorates Experimental Alcoholic Liver Disease by Modulating Oxidative Stress

**DOI:** 10.1155/2017/4287890

**Published:** 2017-12-31

**Authors:** He Peiyuan, Hou Zhiping, Song Chengjun, Wang Chunqing, Li Bingqing, Mustapha Umar Imam

**Affiliations:** ^1^Department of Gastroenterology, The Affiliated Hospital of Chengde Medical University, Chengde, Hebei 067000, China; ^2^Department of Pathology, Chengde Medical University, Chengde, Hebei 067000, China; ^3^Precision Nutrition Innovation Center, School of Public Health, Zhengzhou University, Zhengzhou 450001, China

## Abstract

The aim of this study was to investigate the hepatoprotective effects of resveratrol in alcoholic liver disease (ALD). Alcohol was administered to healthy female rats starting from 6% (v/v) and gradually increased to 20% (v/v) by the fifth week. After 16 weeks of intervention, liver enzymes (aspartate aminotransferase [AST] and alanine aminotransferase [ALT]) were analyzed using a chemistry analyzer, while hepatic antioxidant enzymes, oxidative stress markers, and caspase 3 activity were assessed using ELISA kits. Furthermore, hepatic CYP2E1 protein levels and mRNA levels of antioxidant and inflammation-related genes were determined using western blotting and RT-PCR, respectively. The results showed that resveratrol significantly attenuated alcohol-induced elevation of liver enzymes and improved hepatic antioxidant enzymes. Resveratrol also attenuated alcohol-induced CYP2E1 increase, oxidative stress, and apoptosis (caspase 3 activity). Moreover, genes associated with oxidative stress and inflammation were regulated by resveratrol supplementation. Taken together, the results suggested that resveratrol alleviated ALD through regulation of oxidative stress, apoptosis, and inflammation, which was mediated at the transcriptional level. The data suggests that resveratrol is a promising natural therapeutic agent against chronic ALD.

## 1. Introduction

 Alcoholic liver disease (ALD) often results from binge overconsumption of alcohol. Epidemiological investigations have shown that, in the developed countries of Europe and America, liver diseases account for a significant cause of morbidity and mortality [1, 2]. ALD is becoming a global problem with increasing cases in the developing countries [3]. ALD encompasses fatty liver, alcoholic hepatitis, and chronic hepatitis with liver fibrosis, which are caused by the toxic effects of the byproducts of alcohol metabolism [4, 5]. Clinical testing has demonstrated that ALD patients normally have protein and/or combined protein-calorie malnutrition [6]. On the other hand, rats fed with corn oil and ethanol showed accelerated liver injury because of the polyunsaturated fatty acids intake [7]. Accordingly, risk factors for the development of ALD, including excessive alcohol intake, high fat diet, and calorie malnutrition, have been adopted in the rat model.

Oxidative stress and inflammation are central to the development and progression of ALD [8, 9], often leading to apoptotic cell death as a result of accumulating metabolic end-products of alcohol. Oxidative stress through an imbalance of prooxidant and antioxidant proteins results in oxidative damage including lipid peroxidation [5]. Inflammation, on the other hand, is often a secondary factor that further promotes hepatic damage [10]. Despite the huge burden of the problem at present, there is a lack of effective therapeutics to protect against ALD. Moreover, available pharmaceutical agents are costly and may have side effects. For this reason, interest has heightened in natural products as sources of cost effective and safer alternative therapies for ALD.

Resveratrol is a polyphenol existing in natural products, especially in grape seed. It is a nutraceutical with wide ranging potential therapeutic actions, including antioxidant [11], anti-inflammation [12], cardioprotective [13], and anticancer [14] activities* in vitro* and* in vivo*. Additionally, results of animal experimentation and clinical trial in nonalcoholic fatty liver disease setting have suggested that resveratrol could reduce hepatic fat accumulation, partly through antioxidant and anti-inflammatory actions [15, 16]. Furthermore, nonalcoholic liver disease shares overlapping mechanisms with ALD suggesting that resveratrol may also be effective on ALD. Moreover, resveratrol has been shown to attenuate subacute ALD via regulation of inflammation and hepatosteatosis [17, 18]. There is still need, however, to determine the effect of resveratrol on chronic ALD and the underlying mechanisms involved. Thus, in this study, we explored the effects of resveratrol supplementation against liver damage caused by chronic alcohol intake with limited high fat diet.

## 2. Materials and Methods

### 2.1. Materials

Rat superoxide dismutase (SOD) and catalase (CAT) enzyme-linked immunosorbent assay (ELISA) kits were purchased from Cell Biolabs (INC. USA). Glutathione peroxidase (GPx) and caspase 3 colorimetric assay kits were purchased from Beyotime (Jiangsu, China). Rat chow was purchased from Specialty Feeds (Glen Forrest, WA, Australia) and other solvents of analytical grade were purchased from Merck (Darmstadt, Germany).

### 2.2. Animals, Diets, and Experimental Design

Thirty-six female Wistar rats weighing approximately 200 g were used for the animal experimentation at the Tianjin Medical University (Tianjin, China). All protocols for animal experimentation and maintenance were conducted in accordance with Chengde Medical University Animal Ethics Committee. The animals were kept in individual cages under controlled temperature (25 ± 2°C) and humidity and a 12 h light/dark cycle. Animals were acclimatized for 2 weeks on standard rat chow ad libitum and free access to water. After that, rats were randomly distributed into three groups of nine animals each: control group, maintained on high fat diet; alcoholic liver fibrosis group, maintained on high fat diet and free access to alcohol; resveratrol group, maintained on high fat diet, alcohol, and resveratrol (250 mg/kg BW/day) [19, 20]. The high fat diet fed to the alcohol and resveratrol groups was based on our previous publication [21] and contained 85% standard rat chow plus 15% corn oil. The ethanol was introduced as 6% (v/v) ethanol-containing water for one week and gradually increased to 9% (v/v) for three days, 12% (v/v) for two weeks, and finally 20% (v/v) from the fifth week onwards. Body weights were monitored weekly and food intake was monitored daily. The alcohol and resveratrol groups were fed with isocaloric high fat diet for 16 weeks. Before sacrifice, the rats were fasted for 12 hours, and blood was collected and stored at −80°C. At sacrifice, portions of the liver tissue were snap-frozen in liquid nitrogen or fixed in 10% neutral formalin for further analyses.

### 2.3. Serum Biochemical Assays

Serum aspartate aminotransferase (AST) and alanine aminotransferase (ALT) analyses were performed at several times during the study using blood collected via cardiac puncture after a 12 h fast. Analyses were performed using analytical kits (Biosino Bio-Technology and Science Inc. Beijing, China) based on manufacturer's instructions on the Hitachi-High-Tech instrument (Hitachi High-Technologies Co. Ltd., Shanghai, China).

### 2.4. Morphological Observation

For light microscopy, the dissected livers were fixed in 10% (v/v) formalin saline, and subsequently processed accordingly. Samples were finally stained with hematoxylin and eosin. To evaluate the liver changes, samples were observed at ×200 magnification, and the scale and degrees of liver injury were assessed by two pathologists. For transmission electron microscopy, 1 mm cubes of rat liver were fixed for 4 h at 0°C in 2.5% glutaraldehyde in 0.1 M sodium cacodylate buffer (pH 7·4), washed overnight, and postfixed in 2% (w/v) osmium tetroxide. The tissue was then stained with 2% (w/v) uranyl acetate, ethanol dehydrated, and finally embedded in resin. Sections were poststained with lead citrate, and observed under the transmission electron microscope (Hitachi H-7650).

### 2.5. SOD, CAT, and GPx Antioxidant Activities

SOD, CAT, and GPx activities of the liver were analyzed using commercial ELISA kits based on manufacturer's instructions. Finally the absorbances were read at the appropriate wavelengths on the Synergy H1 Hybrid Multimode Microplate Reader (BioTek, Winooski VT, US), and the results were calculated from the corresponding standard curves (SOD, *y* = 0.319*x* − 0.126, *r*^2^ = 0.998; CAT, *y* = 0.279*x* − 0.300, *r*^2^ = 0.995; GPx: *y* = 0.246*x* − 0.107, *r*^2^ = 0.996).

### 2.6. Thiobarbituric Acid Reactive Substances (TBARS) Assay

Liver samples were homogenized in PBS (50 mg/50 *μ*l PBS) and added to a mixture of 0.25 N HCl, 15% TCA, and 0.375% TBA. The samples were then incubated at 100°C for 10 min, after which they were centrifuged at 3000 rpm for 15 min. Finally, the absorbance of supernatants was read at 540 nm using the Synergy H1 Hybrid Multimode Microplate Reader (BioTek, Winooski VT, US). Tetramethoxypropane (TMP) was used as the standard (*y* = 0.115*x* − 0.266, *r*^2^ = 0.983).

### 2.7. CYP2E1 Western Blotting

Liver samples were homogenized in radio-immune precipitation assay buffer (RIPA) with protease inhibitors. The homogenate was centrifuged at 10,000*g* for 20 min at 4°C. Then, 30 mg protein, determined by the bicinchoninic acid (BCA) protein assay kit, was loaded per well onto a 10% resolving gel and 4% stacking gel. Proteins were transferred to polyvinylidene difluoride (PVDF) membranes and incubated with primary antibody CYP2E1 (1 : 500 dilution, Abnova, Taipei, Taiwan) at 4°C overnight. The horseradish peroxidase- (HRP-) conjugated secondary antibody (Abnova, Taipei, Taiwan) was then added for 1 h at room temperature. The membrane was stripped once for 20 min with stripping buffer and then reprobed with first/secondary antibody as described above for *β*-actin (Sigma, St. Louis, MO, USA) which was used as a loading control. Bands were visualized using 3,3′-diaminobenzidine (DAB) kit (Nacalai Tesque, Inc. Kyoto, Japan). The relative intensities of the immunoreactive bands were captured using a Molecular Imager, ChemiDoc XRS + System (Bio-Rad, Hercules, CA) and quantified with Quantity One Analysis Software, Version 4.6.4 (Bio-Rad, Hercules, CA). The results were expressed as the ratio of protein to *β*-actin.

### 2.8. Liver Caspase 3 Activity

Caspase 3 Activity Assay Kit was used to quantify hepatic caspase 3 activity according to manufacturer's instructions (Beyotime, Jiangsu, China). The absorbances were read at 405 nm on the Synergy H1 Hybrid Multimode Microplate Reader (BioTek, Winooski VT, US).

### 2.9. Quantitative RT-PCR

The total RNA was extracted from rat hepatic tissue using the total RNA extraction kit (Sangon Biotech, Shanghai, China), while reverse transcription was performed using the TaqMan Reverse Transcription Kit according to the manufacturer's instructions. Primer sequences (Table 2) were designed on the National Center for Biotechnology Information (NCBI) website and purchased from Sangon Biotech (Shanghai, China). Quantitative PCR was performed using SYBR Green PCR Master Mix (Qiagen, Inc., Valencia, CA) in a Thermal Cycler (Bioer Technology, Germany). Furthermore, the results were normalized to beta-actin expression and analyzed based on the manufacturer's instructions.

### 2.10. Statistical Analysis

Data were analyzed by one-way ANOVA with Tukey's HSD test, *T* test, Pearson correlation, and linear regression analysis using the Statistical Analysis System (SAS Institute, Cary, NC, USA). Results are expressed as the mean and SEM. *P* ≤ 0.05 was taken as the level of statistical significance.

## 3. Result

### 3.1. Ethanol Intake, Food Intake, and Body Weight

As can be recalled, ALD often results from alcohol overconsumption. Alcohol metabolism byproducts can propagate liver disease through steatohepatitis, fibrosis, cirrhosis, liver failure, and/or hepatocellular carcinoma [10]. At the beginning of the intervention, no significant differences in weights were observed among all the groups (Table 1). At the end of 12 weeks of intervention, however, the control group was significantly different from the alcohol and resveratrol groups, while, after 16 weeks, the body weights of the resveratrol group were increased and significantly different compared to the alcohol group, although they were still lower than those of the control group. In the ALD-inducing period, there was no significant difference in food and alcohol intake between the alcohol and resveratrol groups. The total food and alcohol intake are shown in Table 1.

### 3.2. Liver Enzymes

**Figure 1:**
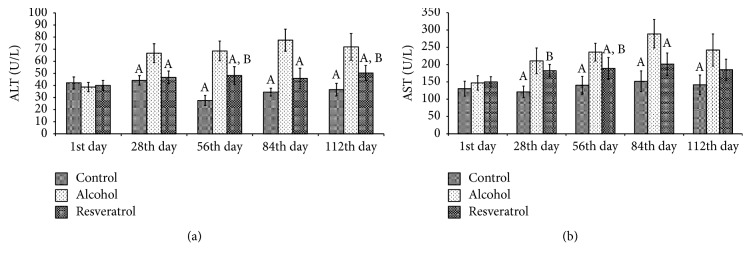
Serum aspartate aminotransferase (AST) and alanine aminotransferase (ALT) levels of the rats during chronic alcohol administration for 16 weeks with resveratrol. Control group received standard rat chow for 16 weeks; alcohol group received high fat diet and alcohol; resveratrol group received high fat diet and alcohol with 250 mg/kg BW/day resveratrol. Data represent mean ± SD of 5 individual values. ^A^*P* < 0.05 versus alcohol group; ^B^*P* < 0.05 versus control group.

There were no significant differences in the ALT and AST among all groups at the beginning of the study (Figure 1; ALT: *P* = 0.4543; AST: *P* = 0.2694). At the end of the fourth week of intervention, however, the alcohol group showed elevated ALT and AST levels in comparison with the other two groups (ALT, *P* < 0.0001; AST, *P* = 0.0004), which progressively increased until the end of the intervention period, suggesting ongoing damage to the liver. The liver enzymes (AST and ALT) in the resveratrol supplementation group were significantly lower than in the alcohol group (*P* < 0.05) after the fourth week.

### 3.3. Morphological Changes

**Figure 2:**
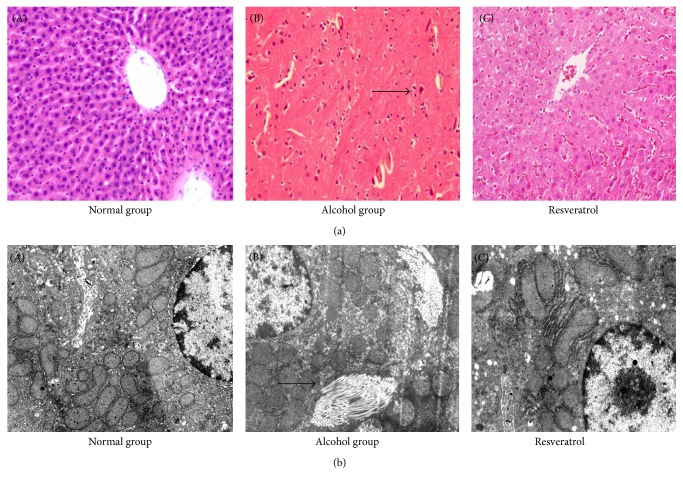
Histological study in the different groups ((A) control, (B) alcohol, and (C) resveratrol). (a) Hematoxylin and eosin staining (light microscope) of liver tissue ×200; (b) Transmission electron microscopy of liver tissue ×15000. Groups are the same as Figure 1.

Morphological changes in the liver are shown in Figure 2. The control group showed liver cells in normal state, with clear cellular boundary, rounded and centrally located nuclear membrane, central lobular vein, and radially arranged and clear sinusoids with no dilatation or congestion (Figure 2(a)(A)). The alcohol group, however, had ballooning of hepatocytes at the acinar zone III, with signs of necrosis (Figure 2(a)(B), shown as arrow), dark double-nucleus hepatocytes, and lymphocyte and neutrophil infiltration. There were also signs of acinar perivascular fibrosis, evidenced by the large numbers of bundles of collagen fibers and a wide range of bridging fibrosis (Figure 2(a)(B), shown as arrow). In some areas, steatosis and inflammation were also visible (Figure 2(a)(B)). Interestingly, the resveratrol group had no bundles of collagen fibers and showed attenuation of the alcohol-induced changes, although rounded fat droplets were still visible in the liver tissue (Figure 2(a)(C)).

No fatty infiltration and bundles of collagen or other abnormal changes were seen in the control group under TEM (Figure 2(b)(A)). In the alcohol group, the Disse space was filled with bundles of collagen, while the microvilli were reduced, and the mitochondria were spherical. There were also fewer and shorter mitochondrial cristae, and the surfaces of the endoplasmic reticulum were smooth and cystic. Bundles of collagen between intercellular spaces and rounded lipid droplets were also visible (Figure 2(b)(B)). Notwithstanding, the resveratrol group significantly attenuated alcohol-induced hepatocellular damage (Figure 2(b)(C)). Accordingly, no deformation of mitochondrial and collagen structures was observed, although few rounded lipid droplets were observed.

### 3.4. Hepatic Antioxidant Capacity

**Figure 3:**
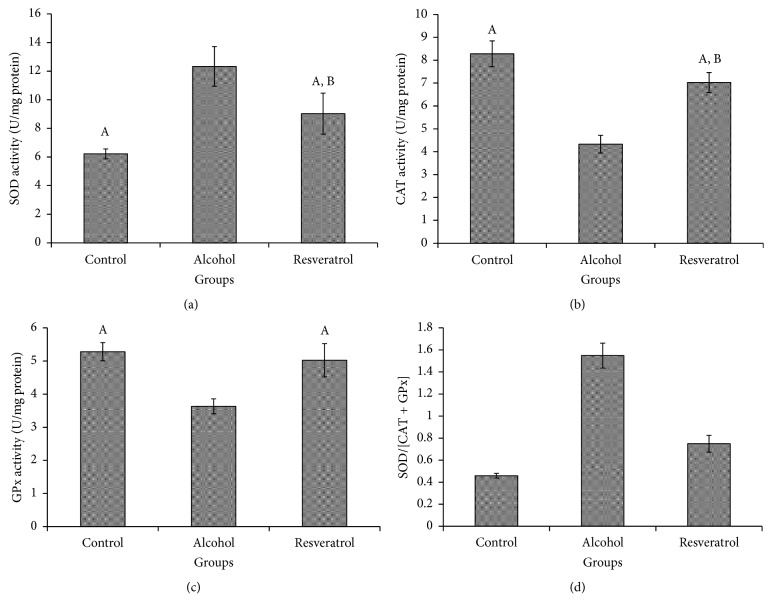
Antioxidant enzymes following chronic alcohol administration for 16 weeks with or without resveratrol supplementation, (a) liver superoxide dismutase (SOD) levels; (b) liver catalase (CAT) levels; (c) glutathione peroxidase (GPx) activity; (d) SOD/[CAT + GPx] level. Groups are the same as Figure 1. Data represent mean ± SD of 5 individual values. ^A^*P* < 0.05 versus alcohol group; ^B^*P* < 0.05 versus control group.

The levels of SOD enzyme activity were assessed in hepatic homogenate. As shown in Figure 3, at the end of the intervention the alcohol group had significantly increased SOD level (*P* < 0.0001; Figure 3(a)), which was attenuated by resveratrol supplementation, although it was not as low as that of the control group (*P* = 0.0067). The alcohol group had significantly lower levels of CAT and GPx activities in comparison with the other groups (CAT, *P* < 0.0001, Figure 3(b); GPx, *P* < 0.0001, Figure 3(c)). Interestingly, the resveratrol-treated group had significantly higher activities of both enzymes (CAT, *P* < 0.0001; GPx, *P* = 0.0001). Additionally, the ratio of SOD/CAT + GPx in the resveratrol group was lower than that of the alcohol group (*P* < 0.0001), although it was not as low as that of the control group (*P* < 0.0001).

### 3.5. Hepatic Oxidative Damage

**Figure 4:**
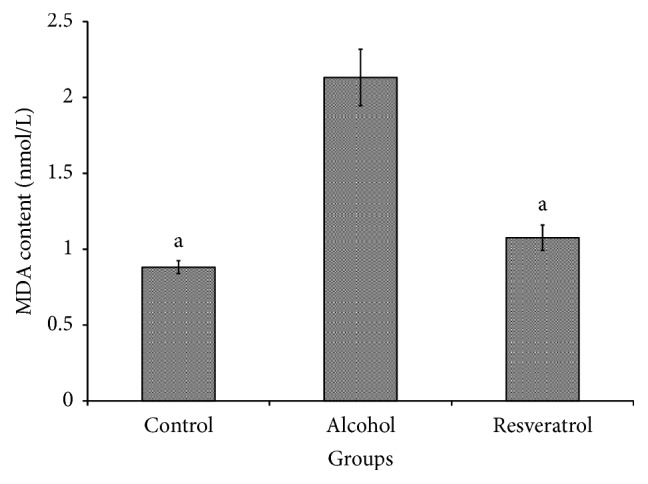
Hepatic malondialdehyde (MDA) at the end of chronic alcohol administration for 16 weeks with resveratrol. Groups are the same as Figure 1. Data represent mean ± SD of 5 individual values. ^a^*P* < 0.05 versus alcohol group.

The hepatic malondialdehyde (MDA) level was estimated as a measure of oxidative damage to the liver. As described in Figure 4, the alcohol group had higher levels of MDA (*P* < 0.0001) in comparison with the other groups, which were not significantly different between each other (*P* = 0.0613).

### 3.6. CYP2E1 Protein Expression

**Figure 5:**
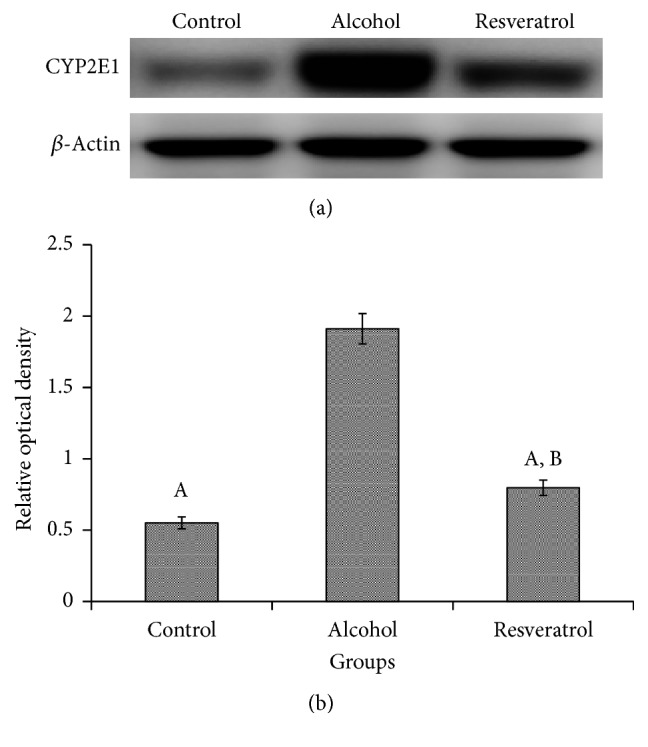
Hepatic CYP2E1 protein expression of rats at the end of chronic alcohol administration after 16 weeks. (a) Representative western blot assay and (b) relative optical density in hepatic homogenate. Groups are the same as Figure 1. Data represent mean ± SD of 5 individual values. ^A^*P* < 0.05 versus alcohol group; ^B^*P* < 0.05 versus control group.

The alcohol group had significantly higher CYP2E1 protein expression in the liver compared to the control and resveratrol groups (*P* < 0.0001; Figure 5). Conversely, the resveratrol group had lower CYP2E1 protein expression in comparison with the alcohol group (*P* < 0.0001).

### 3.7. Hepatic Caspase 3 Activity

**Figure 6:**
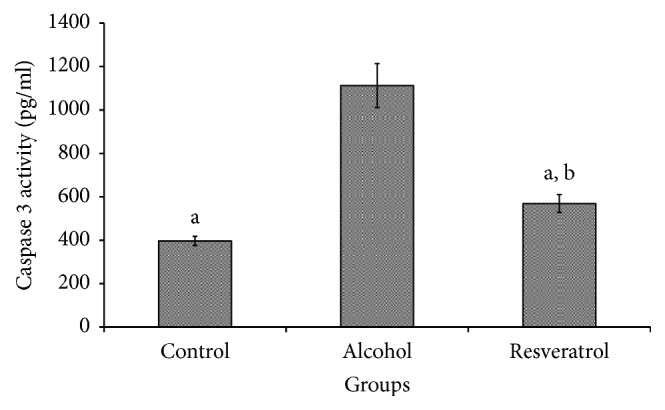
Hepatic caspase 3 protein expression of rats at the end of chronic alcohol administration after 16 weeks. Groups are the same as Figure 1. Data represent mean ± SD of 5 individual values. ^a^*P* < 0.05 versus alcohol group; ^b^*P* < 0.05 versus control group.

Cleaved caspase 3 was upregulated in the alcohol group in comparison with the other groups (*P* < 0.0001; Figure 6). Resveratrol supplementation decreased cleaved caspase 3 activity in comparison with the alcohol group (*P* < 0.001), although it was not as low as that of the control group.

### 3.8. mRNA Levels of Hepatic Antioxidant and Inflammation-Related Genes

**Figure 7:**
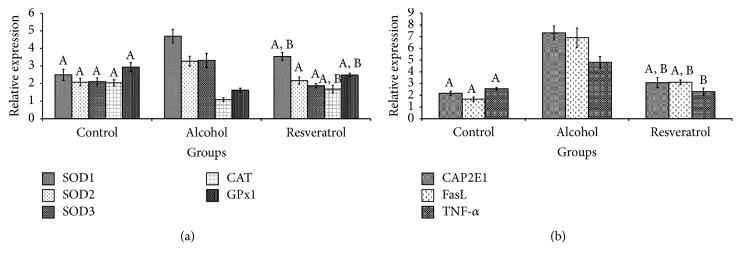
Hepatic mRNA levels of SOD1, SOD2, SOD3, CAT, GPx1, CYP2E1, FasL, and TNF-*α* genes at the end of chronic alcohol administration after 16 weeks. Groups are the same as Figure 1. Data represent mean ± SD of 5 individual values. ^A^*P* < 0.05 versus alcohol group; ^B^*P* < 0.05 versus control group.

Figure 7(a) shows the effects of the interventions on the expression of antioxidant genes (SOD1, SOD2, SOD3, CAT, and GPx). In the present study, alcohol upregulated SOD genes (SOD1, *P* < 0.0001; SOD2, *P* < 0.0001; SOD3, *P* < 0.0001) and decreased the expression of the CAT and GPx genes (CAT, *P* < 0.0001; GPx, *P* < 0.0001). Interestingly, resveratrol supplementation attenuated the alcohol-induced mRNA changes (SOD1, *P* = 0.0004; SOD2, *P* < 0.0001; SOD3, *P* < 0.0001; CAT, *P* = 0.0003; GPx, *P* < 0.0001). Additionally, significant differences were observed in mRNA levels of SOD and GPx genes between the control and resveratrol groups (*P* = 0.0003 and *P* = 0.0073, resp.). Moreover, the mRNA levels of inflammation-related genes (CYP2E1, FasL, and TNF-*α*) were significantly higher in the alcohol group in comparison with the other groups (CAP2E1, *P* < 0.0001; FasL, *P* < 0.0001; TNF-*α*, *P* < 0.0001), although the levels in the control group were significantly lower than in the resveratrol group except for TNF-*α* mRNA expression (*P* = 0.1286).

## 4. Discussion

In the present study, ALD was established using chronic alcohol administration with high fat diet consumption, which is known to induce hepatic steatosis, inflammation, and hepatocyte ballooning, similar to the features of human ALD [22]. Accordingly, signs of incipient fibrosis, worsening of liver function and antioxidant status, and increased inflammatory markers and caspase 3 expression were indicative of successful induction of ALD in the alcohol group.

The effects of resveratrol on ALD were assessed, including mechanistic bases for such effects. In the past, the protective role of resveratrol against liver damage has been predominantly attributed to antioxidant and anti-inflammatory activities. However, both inflammation and oxidative stress are largely secondary effects of liver damage. Therefore, we sought to establish whether resveratrol exerted any direct effect on the major enzymes in the alcohol metabolism pathway. The elimination of alcohol is primarily dependent on the oxidation of alcohol and its metabolic byproducts by CYP2E1, which produces toxic byproducts, like hydroxyethyl, superoxide anion, and hydroxyl radicals [23, 24]. Interestingly, we observed that resveratrol could attenuate CYP2E1 activity and protein levels via western blotting assay. The present findings appear to be consistent with the benefits of resveratrol on suppression of CYP2E1 observed in other liver damage disease models because it is also involved in the metabolism of other toxins in addition to alcohol [25].

On the basis of observations that ethanol administration yields increased amounts of free radicals and hydrogen peroxide, one would logically predict a compensatory increase in enzyme activity to overcome oxidative stress. Moreover, oxidative stress is a well-established factor in the progression of liver damage due to alcohol intake and a high fat diet. In ALD, the byproducts of dysregulated alcohol and lipid metabolism result in the excessive production of free radicals, which leads to oxidative stress [26, 27]. ALD has been demonstrated to be more severe in mice lacking specific antioxidant enzymes [28, 29], underpinning the central role of oxidative damage in ALD and the importance of antioxidants in attenuating such effects. Additionally, chronic alcohol administration typically reduces the antioxidant enzyme activities, as indicated in the present study by the reduced SOD, CAT, and GPx activities. Insufficient antioxidant activity allows free radicals to accumulate and bind to unsaturated fatty acids in cell membranes, which causes lipid peroxidation [30]. Kessova et al. [31] reported that the induction of steatosis after chronic alcohol administration is more severe in Cu/Zn SOD knockout mice. The increased CAT and GPx activities, with lower SOD activity in the resveratrol group in comparison with the alcohol control group, is suggestive of an antioxidative mechanism in attenuation of alcohol-induced oxidative damage by resveratrol. This is further supported by the reduced oxidative stress (MDA) and improved liver function (AST and ALT) in the resveratrol group. There are divergent views on the significance of the absolute values of antioxidant enzyme activities as a reflection of their effects [32, 33], in which case SOD/CAT + GPx ratio has been used [34, 35]. The data again suggested that resveratrol modulated antioxidant enzymes, which may have been the basis for the improved attenuation of oxidative stress and ultimately ALD. Additionally, increased caspase 3 activation in the alcohol group may have been mediated through increased oxidative stress. Moreover, oxidative stress and apoptosis are reported to share common pathways, and the end result of oxidative stress in cells is often apoptotic death [36, 37]. This is supported by the potentiation of antioxidant enzymes and attenuation of both MDA and caspase 3 by resveratrol in this study.

It is well documented that transcriptional regulation of metabolic processes precedes any biochemical changes, and in the present study transcriptomic regulation of antioxidant and inflammation-related genes were evaluated. The data showed that the effects of resveratrol may have been mediated at the transcriptional level. Accordingly, the effects of resveratrol may be long lasting since transcriptional regulation is reported to produce longer lasting effects than biochemical changes alone [38, 39]. Such nutrigenomic regulation may mean that resveratrol may be a more effective alternative than palliative pharmaceuticals presently in use for ALD.

The findings from this study mirror those of previous studies that have shown the beneficial effects of resveratrol on ALD [15, 16]. However, our data adds to the existing knowledge on how resveratrol protects the liver against alcohol damage. Moreover, the duration of our study (16 weeks) mimics that of chronic alcohol exposure more than the previous studies which lasted between 4 and 6 weeks. Similarly, while the previous studies have shown that resveratrol attenuates ALD through regulating inflammation and hepatic lipid metabolism, our data shows that multiple mechanisms, including the modulation of oxidative stress, inflammation, and apoptosis, may be involved in the long term effect of resveratrol against ALD. Another study [19] had demonstrated that resveratrol could attenuate alcohol-induced oxidative stress similar to what was observed in this study. However, the dose used in that study (5 g/kg body weight) calls the reliability of the findings into question. Crowell et al. [20] have demonstrated that doses of resveratrol above 300 mg/kg in rodents are toxic, suggesting that the results from the study by Kasdallah-Grissa et al. [19] may have been influenced by toxicity from resveratrol.

Taken together, the present findings indicate that resveratrol may have alleviated ALD through alleviation of oxidative stress, apoptosis, and inflammation, which were mediated at the transcriptional level. Moreover, resveratrol affected multiple processes and pathways suggesting its effectiveness in alleviating ALD.

## 5. Conclusions

These results demonstrated that resveratrol (250 mg/kg BW/day) effectively attenuated alcohol-induced hepatotoxicity in female Wistar rats. The hepatoprotective effects of resveratrol were mediated by modulating multiple pathways including oxidative stress, inflammation, and apoptosis. The evidence obtained in this study suggested the effectiveness of resveratrol as a natural therapy for ALD.

## Figures and Tables

**Table 1:** Body weights and food intake in alcoholic liver disease rat model after 16 weeks of intervention.

Rat groups	Control group	Alcohol group	Resveratrol group
Body weight at beginning (g)	188.89 ± 12.24	185.45 ± 3.21	190.21 ± 6.31
Body weight at end of 4th week (g)	207.34 ± 13.55	204.95 ± 14.50	202.85 ± 12.18
Body weight at end of 8th week (g)	232.40 ± 17.45	221.91 ± 18.70	222.65 ± 14.38
Body weight at end of 12th week (g)	255.86 ± 26.74	229.74 ± 23.87^a^	232.11 ± 4.8^a^
Body weight at end of 16th week (g)	298.6 ± 35.91	217.85 ± 25.16^a,b^	254.97 ± 24.99^a^
Total food intake (Kcal./kg/day)	155.83 ± 8.56	64.56 ± 4.43	72.21 ± 5.66
Total alcohol intake (g/kg/day)	NA	8.23 ± 2.14	7.89 ± 3.31

Values are mean ± SD, *n* = 9. Control group received standard rat chow for 16 weeks; alcohol group gained high fat diet and alcohol liquid; resveratrol group received high fat diet and alcohol liquid with 250 mg/kg BW/day resveratrol. ^a^*P* < 0.05 versus normal control group; ^b^*P* < 0.51 versus alcohol group.

**Table 2:** Names, accession number, and primer sequences used in the study.

Gene name	Accession number	Forward sequence	Reverse sequence
*Kan(r)* ^*∗∗*^			
Beta-actin^*∗*^	NM_031144	AGGTGACACTATAGAATA GGCATCCTGACCCTGAAGTA	GTACGACTCACTATAGGGA AGACGCAGGATGGCATGAG
SOD1	NM_017050	AGGTGACACTATAGAATA TCAATATGGGGACAATACAC	GTACGACTCACTATAGGGA TACTTTCTTCATTTCCACCTT
SOD2	NM_017051	AGGTGACACTATAGAATA TGTATGAAAGTGCTCAAGAT	GTACGACTCACTATAGGGA GCCCTCTTGTGAGTATAAGT
SOD3	NM_012880	AGGTGACACTATAGAATA TCGAACTACTTTATGCCC	GTACGACTCACTATAGGGA GAAGACAAACGAGGTCTCTA
CAT	NM_012520	AGGTGACACTATAGAATA ACTGCAAGTTCCATTACAAG	GTACGACTCACTATAGGGA GTTCAACTTCAGCAAAATAAT
GPx	NM_001039848	AGGTGACACTATAGAATA GTGCCAGAGCCGGGG	GTACGACTCACTATAGGGA GGTCTTGCCTCACTGGGA
CYP2E1	NM_031543	AGGTGACACTATAGAATA AGAGGGAATTGGATC	GTACGACTCACTATAGGGA TGCCTGGGATCAGGTTA
FasL	NM_010177	AGGTGACACTATAGAATA GTGCCCCGGAATTGGG	GTACGACTCACTATAGGGA GGGCTCAATTCCCAA
TNF-*α*	NM_001024447	AGGTGACACTATAGAATA GCCAAGGGGTTGGGAA	GTACGACTCACTATAGGGA GGGACTTTGGGGGAAA

^*∗*^Housekeeping gene. Underlined sequences are left and right universal left and right sequences (tags). ^*∗∗*^Internal control supplied by Beckman Coulter Inc. (Miami, FL, USA) as part of the GeXP kit. RT conditions were 48°C for 1 min; 37°C for 5 min; 42°C for 60 min; 95°C for 5 min and then held at 4°C. PCR conditions were initial denaturation at 95°C for 10 min, followed by two-step cycles of 94°C for 30 sec and 55°C for 30 sec, ending in a single extension cycle of 68°C for 1 min. IDE: insulin degrading enzyme; LRP: low density lipoprotein receptor-related protein 1; SOD: superoxide dismutase; CAT, catalase; PSEN: presenilin; APP: amyloid precursor protein. Gapdh: glyceraldehyde-3-phosphate dehydrogenase; Kan(r): kanamycin resistance.

## Abbreviations

ALD: Alcoholic liver disease
SOD: Superoxide dismutase
CAT: Catalase
GPx: Glutathione peroxidase
FasL: Fas ligand
CYP2E1: Cytochrome P450 2E1
TNF: Tumor necrosis factor
ALT: Alanine aminotransferase
AST: Aspartate aminotransferase.
